# Transplant centers’ prophylaxis and monitoring strategies: a key determinant of current herpes and polyomavirus incidences – results from the DZIF kidney transplant cohort

**DOI:** 10.1186/s12882-025-04084-5

**Published:** 2025-04-30

**Authors:** Claudia Sommerer, Iris Schröter, Katrin Gruneberg, Daniela Schindler, Christian Morath, Lutz Renders, Gunilla Einecke, Martina Guthoff, Uwe Heemann, Paul Schnitzler, Martin Zeier, Thomas Giese, the Transplant Cohort of the German Center for Infection Research (DZIF Transplant Cohort) Consortium, Christine S. Falk, Nele Kanzelmeyer, Anette Melk, Thomas F. Schulz, Susanne Delecluse, Philipp Ehlermann, Uta Merle, Burkhard Tönshoff, Joachim Andrassy, Martin Hildebrandt, Michael Neuenhahn, Tina Ganzenmüller, Thomas Iftner, Peter Lang, Berit Lange, Carolina Klett-Tammen, Bärbel Fösel, Thomas Illig

**Affiliations:** 1 Nephrology, University Hospital Heidelberg, Im Neuenheimer Feld 162, D-69120 Heidelberg, Germany; 2 Department of Nephrology, Klinikum rechts der Isar of the Technical University Munich, Munich, Germany; 3 Department of Nephrology, Hannover Medical School, Hannover, Germany; 4 Department of Nephrology and Rheumatology, University Medical Centre Göttingen, Göttingen, Germany; 5 Department of Diabetology, Endocrinology, Nephrology, University Hospital Tuebingen, Tuebingen, Germany; 6 Department of Infectious Diseases, University Hospital Heidelberg, Heidelberg, Germany; 7 Department of Immunology, University Hospital Heidelberg, Heidelberg, Germany; 8 German Centre for Infection Research (DZIF), Heidelberg, Germany

**Keywords:** Renal transplantation, Infection, CMV, BKV, Prophylaxis, Cohort study

## Abstract

**Background:**

Herpes- and polyomaviruses are major opportunistic pathogens after renal transplantation. Despite established guidelines, there is limited data on transplant centers’ prophylaxis and monitoring strategies and centers’ adherence to these guidelines and their impact on infection rates and patient outcomes.

**Methods:**

This multicenter cohort study, conducted by the German Center for Infection Research, included 1035 kidney transplant recipients from five centers (01/2014–02/2021), focusing on herpes- and polyomavirus viremia within the first year and adherence to prophylaxis strategies.

**Results:**

Among 1035 recipients, 26.6% developed herpes- or polyomavirus viremia, predominantly Cytomegalovirus (CMV, 14.3%) and BK-virus (BKV, 13.2%). BKV monitoring frequency was below guideline recommendations. Deviations from guidelines were most common in CMV D-/R- (34.6% with prophylaxis) and D−/R + groups (37.3% without prophylaxis), doubling CMV-incidence in D−/R+ (28.9% vs. 12.5%, *p* < 0.01). In D+/R − group, six-month-prophylaxis reduced CMV-incidence compared to three months (22.5% vs. 38.4%, *p* < 0.01). Breakthrough-viremia was most commonly observed in D+/R − recipients who received a six-month-prophylaxis. Overall, viremia was associated with higher incidence of acute rejection (31.9% vs. 17.6%, *p* < 0.01), with most CMV-viremias occurring after rejection. CMV-viremia was associated with a higher risk of bacterial infection (HR = 1.77, [1.03;3.02]). Other herpesviruses were associated with a quadrupled risk for fungal infection (HR = 4.34, [1.03;18.30]) and the non-administration of CMV-prophylaxis (HR = 0.22, [0.11;0.47]). Graft survival and mortality were unaffected within the first year.

**Conclusion:**

Clinical variability in guideline implementation drives high herpes- and polyomavirus infection rates with suboptimal outcomes. Future guidelines should focus on differentiated risk stratification to address breakthrough, post-prophylaxis, and post-rejection CMV, and include protocols for the early detection of secondary infections.

**Supplementary Information:**

The online version contains supplementary material available at 10.1186/s12882-025-04084-5.

## Background

The survival of renal allografts has been improved markedly by immunosuppressive therapy, such as calcineurin inhibitors. However, immunosuppression is accompanied by opportunistic infections [1], which are a leading cause of death with a functioning graft [2]. The major pathogens causing transplant loss after kidney transplantation are the herpesvirus cytomegalovirus (CMV) and the polyomavirus BK-virus (BKV). Primary BKV infection usually occurs in childhood and persists latently over years [3, 4]. Immunocompromised individuals are at risk of reactivation and of primary infection, either from the donor graft or from other people [5]. In recent years, an increase in BKV has been observed in renal transplant recipients [6]. BKV infection causes BKV-associated nephropathy (BKVAN) in around 10% of patients and allograft loss in around 50% of patients [6]. Similarly, CMV infection occurs frequently after transplantation and can lead to multiorgan diseases, diabetes, and cardiovascular morbidity and can reduce patient and graft survival, despite efficacious anti-CMV prophylaxis being available [5, 7]. Treatment is often complicated by side effects such as neutropenia or nephrotoxicity [8].

Concerning prophylaxis strategies and monitoring, existing guidelines are often weak. For example, KDIGO 2009 guidelines suggest BKV screening of kidney transplant recipients at least monthly for the first 3–6 months after transplantation and every 3 months until the end of the first post-transplant year. However, this is only a suggestion on a 2D level with very low evidence. KDIGO 2009 also recommends CMV prophylaxis with oral ganciclovir or valganciclovir for at least 3 months after transplantation, except when donor and recipient both have negative CMV serologies on a level 1B recommendation with moderate evidence [9]. Studies in the high-risk population with a CMV-positive donor (D+) and CMV-negative recipient (R-) have shown that antiviral chemoprophylaxis reduces the incidence of CMV disease by about 60%, but prophylaxis has also been shown to be effective in recipients at moderate risk for CMV disease (e.g. CMV D+/R+, or D-/R+)[10]. The use of antiviral chemoprophylaxis has also demonstrated to reduce the incidence of CMV-associated mortality, all-cause mortality, as well as clinically important disease due to opportunistic infections [11].

Although the risk factors of herpes- and polyomaviruses have been well studied [7, 12, 13], the impact of non-adherence to prophylaxis and monitoring recommendations by transplant centers and differing prophylaxis strategies in real-world clinical practice on incidences and transplant outcomes have not yet been evaluated. Efficiency of existing guidelines can only be properly assessed when we understand how guidelines are actually implemented. Another controversial topic is whether CMV prophylaxis or CMV replication promotes or triggers BKV [11, 13]. Co-viremia has been shown to severely affect renal allograft function [12, 14].

The primary aim of this study was to assess the incidences of herpes- and polyomaviruses infection in a large prospective multicenter renal transplant cohort, and to determine to which extend these are affected by transplant centers’ prophylaxis and monitoring strategies as well as adherence to existing guidelines as the KDIGO 2009 guideline [9]. Secondary aims were to assess risk factors, outcomes and associations with rejections episodes and other infections.

## Methods

### Study design

A multicenter prospective kidney transplant cohort study was conducted by the German Center for Infection Research (Deutsches Zentrum für Infektionsforschung; DZIF) and involved five of the largest transplant centers in Germany (University Hospital Hannover, University Hospital and Renal Center Heidelberg, TU Munich, LMU Munich, and University Hospital Tuebingen) [15]. The DZIF Transplant Cohort study design has been described elsewhere in detail [15], and allows the expertise of a multidisciplinary scientist team, including nephrologists, surgeons, virologists, and immunologists, to be shared.

The study was conducted in accordance with the Declaration of Helsinki and the International Conference on Harmonization Guidelines for Good Clinical Practice and was approved by the Ethics Committees of participating centers (Hannover Medical School Nr 6534, Medical Faculty of the University of Heidelberg Nr S-585/2013, Medical Faculty of the TU Munich Nr 5926/13, LMU Munich Nr 380 − 15, University Hospital Tuebingen Nr327/2014BO1). Written informed consent was obtained from all participants.

### Setting & study cohort

Inclusion criteria were adult DZIF participants undergoing renal transplantation or simultaneous pancreas-kidney transplantation between January 2014 and February 2021. Follow-up visits occurred at 3, 6, 9, and 12 months after transplantation, and in case of infectious complications.

Patient, clinical, and laboratory data were extracted from patient files at baseline and at each visit and recorded in a web-based database by trained medical professionals.

The immunosuppressive regimen was similar in all centers and consisted of a calcineurin inhibitior (tacrolimus (Tac) or ciclosporine A (CsA)), mycophenolate sodium or mycophenolate mofetil, and methylprednisolone. Target trough (C0) levels for Tac were 6–9 ng/ml at month 1, 5–8 ng/ml at month 3, and 4–7 ng/ml thereafter, for CsA 150–180 ng/ml, 100–150 ng/ml, and 80–120 ng/ml, respectively. Mycophenolic acid (MPA) was used either as enteric coated mycophenolate sodium (1.44 g/day) or mycophenolate mofetil (2 g/day). Depending on immunological risk, either basiliximab or thymoglobuline was used for induction therapy. Immunized transplants were defined as re-transplantation or the presence of high donor-specific anti-HLA antibodies (DSA), and these patients were grouped separately. For AB0-incompatible transplants, induction therapy typically included additional immunosuppressive treatments, such as immunoadsorption with or without rituximab.

Allograft function was monitored by measuring serum creatinine levels at each visit. The estimated glomerular filtration rate (eGFR) was calculated using the CKD-epi formula [16]. Biopsies were taken if allograft rejection or BKVAN was suspected and were tested according to the 2005 Banff criteria by an independent local pathologist [17]. Protocol biopsies were excluded form analyses. Borderline and T-cell-mediated rejections were treated with bolus methylprednisolone, given intravenously for at least 3 days. If the patient was unresponsive to this treatment, thymoglobuline was administered. The treatment for acute antibody-mediated rejection was primarily based on center-specific protocols and clinical judgment. It typically included plasmapheresis, rituximab (RTX) or intravenous immunoglobulin (IVIG).

### Virus prophylaxis, monitoring and reactivation management

As prospective observational cohort study with a protocol set-up starting 2012, recommendations on virus prophylaxis, monitoring, reactivation management, and therapy were based on KDIGO 2009 guidelines [14]. CMV prophylaxis with valganciclovir adapted to renal transplant function was recommended for at least 3 months for CMV IgG-positive donors (D+)/CMV IgG-negative recipients (R−), D+/CMV IgG-positive recipients (R+), and CMV IgG-negative donors (D−)/R + as well as for three months in case of T-cell-depleting induction therapy and six weeks after treatment with a T-cell-depleting antibody. No herpesvirus prophylaxis was recommended for D−/R − patients.

There was no recommendation for special Herpes simplex virus (HSV) and Varicella zoster virus (VZV) prophylaxis in the absence of CMV prophylaxis. CMV and BKV viral load were monitored by real-time polymerase chain reaction (PCR) in plasma. During CMV prophylaxis, as well as after its discontinuation, routine monitoring for asymptomatic viremia was dependent on the center’s recommendations (Table S1) and on clinical judgment.

Monthly monitoring of BKV viral load was recommended for the first 3–6 months after transplantation and every 3 months thereafter until the end of the first year.

Additional testing was suggested in case of an unexplained raise of serum creatinine and after treatment for acute rejection. Other viral loads were assessed in case of clinical suspicion.

Non-adherence was defined as deviation from KDIGO 2009 guidelines based on documented prophylaxis and center protocol. The term “missing prophylaxis” refers specifically to cases where prophylaxis was not administered.

Viremia was defined as a period during which a patient tests positive for the presence of a specific virus in their blood, with viral load exceeding the clinically relevant threshold. For patients who experience multiple detectable viral loads above the threshold during the observation period, each occurrence is considered a separate viremia. Clinically significant viremia as sign of active infection was defined as above 10,000 copies/mL for BKV and 1000 IU/mL for CMV and Epstein-Barr-virus (EBV). Because there are no cut-off recommendations for Human Herpes virus-6 (HHV-6), Human Herpes virus-7 (HHV-7), Human Herpesvirus-8 (HHV-8) or JC-Virus (JCV) viremia, detection of these viruses in plasma was defined as active replication and included in the analyses. CMV disease including CMV syndrome [18] and tissue-invasive disease was diagnosed by significant viremia and typical clinical symptoms and/or organ specific diagnostics. Superficial HSV and VZV infection was diagnosed by clinical presentation followed by qPCR of lesion fluids or mucosae. Tissue-invasive/end-organ diseases were diagnosed by histopathological analysis [19]. Patients with significant viremia were regularly monitored by qPCR at least until the first negative result. Patients with CMV and BKV viremia, either simultaneously or at different time points, were defined as having CMV/BKV-co-viremia.

### Outcomes

The primary outcome was the first detection of CMV and BKV viremia above the clinically relevant cut-off, considering the centers’ prophylaxis and monitoring strategy. Additionally, the incidence of CMV disease and syndrome was assessed. Secondary outcomes included the first detection of EBV above the relevant cut-off, HHV-6, HHV-7, HHV-8- and JCV-viremia, and the first clinical presentation of HSV and VZV infection as well as CMV/BKV-co-viremias, bacterial and fungal infection, biopsy-proven acute rejection, graft loss, and death. Bacterial infections were included if confirmed by positive microbial cultures from body fluids or definitive clinical signs requiring antimicrobial treatment. Fungal infections were included if they were suspected to be invasive, based on a combination of clinical presentation, imaging, positive laboratory findings (e.g., fungal antigens or DNA in clinical samples), cultures, or histopathological examination, depending on the type of fungi and the site of infection.

### Statistical analysis

Analyses were performed using IBM SPSS Statistics Version 28.0 (SPSS Inc. Chicago, IL, USA) and Addinsoft XLSTAT Version 2022.2.1 (New York, USA) for Mac OS X. Results were expressed.

as means with standard deviations (SD) or as medians with interquartile ranges (IQR). Continuous variables were compared using the Mann–Whitney U test or Student’s t-test, and categorical variables were compared using the Chi-Square test or Fisher’s exact test. Statistical significance was defined as a p value < 0.05. Time of observation was calculated as the time between transplantation and 365 days thereafter. Cumulative incidence rates with 95% confidence intervals (CIs) were calculated as the percentage of affected patients during the observation period, censoring for all competing events (e.g., death, graft loss). Cox proportional hazard regression analyses were performed to identify risk factors for viremia and to evaluate associations between viremia and rejections episodes. To account for the dynamic nature of clinical events, time-dependent covariates were used. These covariates allowed the model to update exposure statuses dynamically, reflecting the temporal sequence of events (e.g., the onset of viremia influencing subsequent rejection risk). Multivariate analyses of all data with a *p* < 0.10 in the univariate analysis were performed to control for cofounding. In cases of overlapping risk factors, the factor considered most influential was included in the final model.

## Results

### Baseline characteristics and overall outcome

A total of 1316 patients were included in the DZIF kidney transplant cohort between January 2014 and February 2021. In the present analyses, 84 patients were excluded due to due to being under 18 years of age,132 patients due to follow-up period of less than one year, and 15 patients due to incomplete or unreliable data (e.g. missing baseline or follow-up data or withdrawal of consent).

Consequently, 1035 recipients were included in the present analyses. – 418 (40.4%) at center 1, 209 (20.2%) at center 2, 167 (16.1%) at center 3, 144 (13.9%) at center 4, and 97 (9.4%) at center 5. The mean age was 51 years, 64.6% were male, and 33.1% received their graft from a living donor (Table 1).

**Table 1:** Demographics of the total patient cohort, and in renal allograft recipients without and with herpes virus or polyoma virus viremia

	Total cohort	No viremia	CMV viremia	Other herpes viruses	BKV viremia	CMV/ BKV co-viremia
**Total number of patients**	**1035**	767	144	31	132	23
**Demographics**						
Age at tx (Mean ± SD, range)	51 ± 14 18–79	51 ± 14 18–78	50 ± 13 19–78	53 ± 12 20–79	52 ± 13 22–79	49 ± 13 25–76
< 50 years	413 (40.0)	309 (40.4)	59 (41.0)	8 (25.8)	49 (37.1)	10 (43.5)
50–65 years	448 (43.4)	318 (41.6)	70 (48.6)	19 (61.3)	66 (50.0)	12 (52.2)
> 65 years	170 (16.5)	138 (18.0)	15 (10.4)	4 (12.9)	17 (12.9)	1 (4.3)
Male gender	654 (64.6)	473 (63.6)	94 (65.2)	23 (74.2)	97 (73.5)	19 (82.6)
**Clinical data**						
**Cause of ESRD**						
Glomerulonephritis	321 (31.5)	224 (29.6)	55 (38.5)	13 (43.3)	43 (33.1)	8 (34.8)
APKD	147 (14.4)	113 (14.9)	16 (11.2)	6 (20.0)	21 (16.2)	5 (21.7)
Diabetes mellitus	105 (10.3)	82 (10.8)	16 (11.2)	3 (15.0)	7 (5.4)	1 (4.3)
Nephrosclerosis	54 (5.3)	46 (6.1)	4 (2.8)	1 (3.3)	3 (2.3)	0 (0.0)
Interstinal Nephritis	32 (3.1)	23 (3.0)	3 (2.1)	0 (0.0)	8 (6.2)	2 (8.7)
Vasculitis and Collagenoses	29 (2.8)	20 (2.6)	4 (2.8)	0 (0.0)	5 (3.8)	0 (0.0)
Urological diseases	22 (2.2)	19 (2.5)	1 (0.7)	1 (3.3)	2 (1.5)	0 (0.0)
Other hereditary diseases	59 (5.8)	41 (5.4)	9 (6.3)	1 (3.3)	12 (9.2)	4 (17.4)
Other	251 (24.6)	190 (25.1)	32 (22.4)	5 (16.7)	29 (22.3)	3 (13.0)
Body mass index (mean ± SD) in kg/m^2^	25 ± 4	26 ± 5	25 ± 4	25 ± 4	25 ± 4	25 ± 4
**Donor characteristics**						
Age group						
< 35	107 (11.0)	86 (12.0)	10 (7.1)	1 (3.3)	11 (8.5)	0 (0.0)
≥ 35 to < 60	459 (47.0)	337 (47.2)	66 (47.1)	19 (63.3)	56 (43.1)	10 (45.5)
≥ 60	411 (42.1)	291 (40.8)	64 (45.7)	10 (61.3)	63 (48.5)	12 (54.5)
Male sex	381 (43.0)	290 (44.3)	46 (37.7)	14 (33.3)	45 (38.8)	7 (36.8)
**CMV serologies**						
D+/ R-	204 (20.9)	126 (17.5)	57 (41.6)	0 (0.0)	33 (25.4)	12 (54.5)
D+/ R+	349 (35.7)	266 (37.0)	46 (33.6)	6 (20.0)	40 (30.8)	6 (27.3)
D-/ R+	201 (20.6)	151 (21.0)	30 (21.9)	7 (23.3)	21 (16.2)	3 (13.6)
D-/ R-	225 (23.0)	176 (24.5)	4 (2.9)	17 (56.7)	36 (27.7)	1 (4.5)
**Type of transplantation**						
Living donation	341 (33.1)	267 (34.9)	30 (21.0)	11 (35.5)	41 (31.3)	3 (13.6)
Pancreas-kidney	56 (5.4)	40 (5.2)	12 (8.3)	1 (3.2)	4 (3.0)	1 (4.3)
AB0-Incompatibility	57 (5.9)	45 (6.4)	6 (4.3)	1 (3.2)	6 (4.6)	1 (4.3)
Previous transplantation	168 (16.2)	122 (15.9)	23 (16.0)	6 (19.6)	24 (18.2)	5 (21.7)
Immunized transplantation	81 (8.3)	49 (6.9)	47 (33.8)	12 (40.0)	13 (10.0)	2 (9.1)
ESP	130 (12.6)	108 (14.0)	11 (7.6)	3 (9.7)	11 (8.3)	0 (0.0)
**Immunosuppression**						
**Induction therapy**						
Basiliximab	863 (83.4)	654 (84.7)	110 (78.6)	24 (77.4)	107 (81.1)	17 (73.9)
Thymoglobuline	172 (16.6)	117 (15.3)	34 (23.6)	7 (22.6)	25 (18.9)	6 (26.1)
Plasmapheresis	141 (13.6)	95 (12.4)	29 (20.1)	3 (9.7)	22 (16.7)	6 (26.1)
Conditioning treatment^†^	285 (28.9)	200 (27.9)	55 (38.5)	13 (41.9)	41 (31.3)	9 (39.1)
**Maintenance therapy at discharge**						
Tacrolimus + MPA/MMF + Steroids	674 (79.1)	472 (77.9)	109 (82.6)	21 (70.0)	102 (86.4)	17 (89.5)
Ciclosporine A + MPA/MMF + Steroids	175 (20.5)	133 (21.9)	23 (17.4)	9 (30.0)	16 (13.6)	2 (10.5)
Tacrolimus + Azathioprine + Steroids	2 (0.2)	2 (0.3)	0 (0.0)	0 (0.0)	0 (0.0)	0 (0.0)
**Antimicrobial Prophylaxis**						
CMV-prophylaxis	772 (75.9)	493 (75.6)	126 (89.4)	12 (40.0)	89 (78.1)	17 (85.0)
**Postoperative variables**						
In-patient stay, (Md, IQR)	17, 12–24	17, 12–24	19, 14–26	17, 14–28	16, 13–22	18, 14–25
Delayed graft function^‡^	195 (19.1)	138 (18.3)	32 (22.2)	7 (22.6)	21 (16.0)	1 (4.3)

Data presented as numbers (%) unless otherwise indicated. Missing values were excluded.

Other herpes viruses included HSV-1 (*n* = 15), HSV-2 (*n* = 6), VZV (*n* = 9), EBV (*n* = 5), HHV-6 (*n* = 1).

Abbreviations: tx = transplantation, ESRD = end-stage renal disease, APKD = autosomal polycystic kidney disease, CMV = Cytomegalovirus, BKV = BK-virus, HSV = Herpes simplex virus, VZV = Varicella zoster virus, EBV = Epstein-Barr-virus, HHV-6 = Human Herpesvirus 6, R+/-=recipient positive/negative, D+/-=donor positive/negative, ESP = Eurotransplant Senior Program, MPA = Mycophenolic acid, MMF = Mycophenolate mofetil; C0 = trough level, CsA = Ciclosporin A, Tac = tacrolimus, SD = standard deviation, Md = median, IQR = interquartile range. Plasmapheresis, Thymoglobuline Rituximab etc. before transplantation

^‡^Need for hemodialysis within the first 7 days post-transplantation

18 (1.7%) died during our observation period, at a mean age of 62 ± 9 years. The predominant cause of death was infection (9/18), primarily invasive fungal diseases. Graft loss was reported in 24 (2.3%) patients after a median of 155 days (IQR = 82–223). The incidence of biopsy-proven acute rejection was 21.4% (Table 2). 290 cases were diagnosed overall (60.0% borderline rejections, 36.9% T-cell-mediated rejections, 2.4% antibody-mediated rejections). After 12 months, mean eGFR was 48.9 ± 19.5 ml/min/1.73m^2^ and 43.4% had an impaired allograft function (creatinine > 1.5 mg/dl; eGFR < 40 ml/min/1.73m^2^).

**Table 2:** Cumulative-incidence-rates and median times to first detection of general outcome and infection data

	Cumulative-incidence-rate (%) [95%-CI]	Incidence (no.)	Median time to first detection (IQR)
General outcome			
Death	1.7 [1.10;2.80]	18	141 (66–275)
Graft loss	2.3 [1.60;3.50]	24	155 (82–223)
Biopsy proven acute rejection (all)	21.4 [18.90;24.00]	218	90 (30–163)
Biopsy proven acute rejection (without borderline)	10.0 [8.30;12.00]	101	133 (77–187)
T-cell mediated rejection	9.5 [7.90;11.50]	97	135 (78–188)
Banff IA	3.5 [2.50;4.80]	34	121 (86–182)
Banff IB	1.2 [0.70;2.20]	12	130 (92–184)
Banff IIA	3.8 [2.70;5.20]	37	135 (77–185)
Banff IIB	1.2 [0.70;2.20]	12	102 (70–168)
Banff III	0.2 [0.10;0.80]	2	143 (139-147)
Antibody-mediated rejection	0.7 [0.03;0.015]	7	189 (100–271)
Borderline rejection	14.2 [12.2;16.5]	147	79 (20–138)
**Infections**			
**Herpes/Polyomaviruses**	26.6 [24.1;29.4]	269	119 (76–188)
**Herpesviruses**	16.7 [14.6;19.2]	169	129 (71–208)
CMV	14.3 [12.3;16.6]	144	138 (77–219)
D+/R-	28.6 [22.9;35.6]	57	119 (90–160)
D+/R+	13.6 [10.4;17.8]	46	176 (117–255)
D-/R+	15.1 [10.8;21.0]	30	131 (56–181)
D-/R-	1.8 [0.7;4.8]	4	94 (38–167)
**Other herpesviruses**	3.0 [2.2;4.3]	31	90 (45–145)
HSV-1	1.5 [0.9;2.5]	15	90 (42–148)
VZV	0.9 [0.5;1.7]	9	93 (53–152)
HSV-2	0.6 [0.3;1.3]	6	90 (60–180)
EBV	0.7 [0.3;1.4]	7	123 (95–158)
HHV-6	0.1 -	1	-
**Polyomaviruses**	13.2 [11.2;15.4]	132	108 (78–174)
BKV	13.2 [11.2;15.4]	132	108 (78–174)
JCV	0.1 [-]	1	-
**Other infections**			
Bacterial infection	41.7 [38.8;44.8]	428	30 (10–85)
Fungal infection	4.7 [3.6;6.2]	48	52 (17–140)

Time to first infection was calculated as time to first detection of viremia via PCR;

Abbreviations: CMV = Cytomegalovirus, D + = Donor positive, R-=Recipient negative, HSV = Herpes simplex virus, VZV = Varicella zoster virus, EBV = Epstein-Barr-Virus, HHV-6 = Human Herpesvirus 6, BKV = BK-Virus, JCV = JC-Virus; 95%-CI = 95%-confidence interval, no. = number, IQR = interquartile range

Within the first year, 269 patients developed 377 cases of herpes- or polyomavirus viremia, (incidence: 26.6%, [95% CI, 24.1; 29.4]). 16.7% (95% CI, 14.6; 19.2) developed at least one episode of herpesvirus viremia (Table 2).

### Herpesviruses

**Fig. 1:**
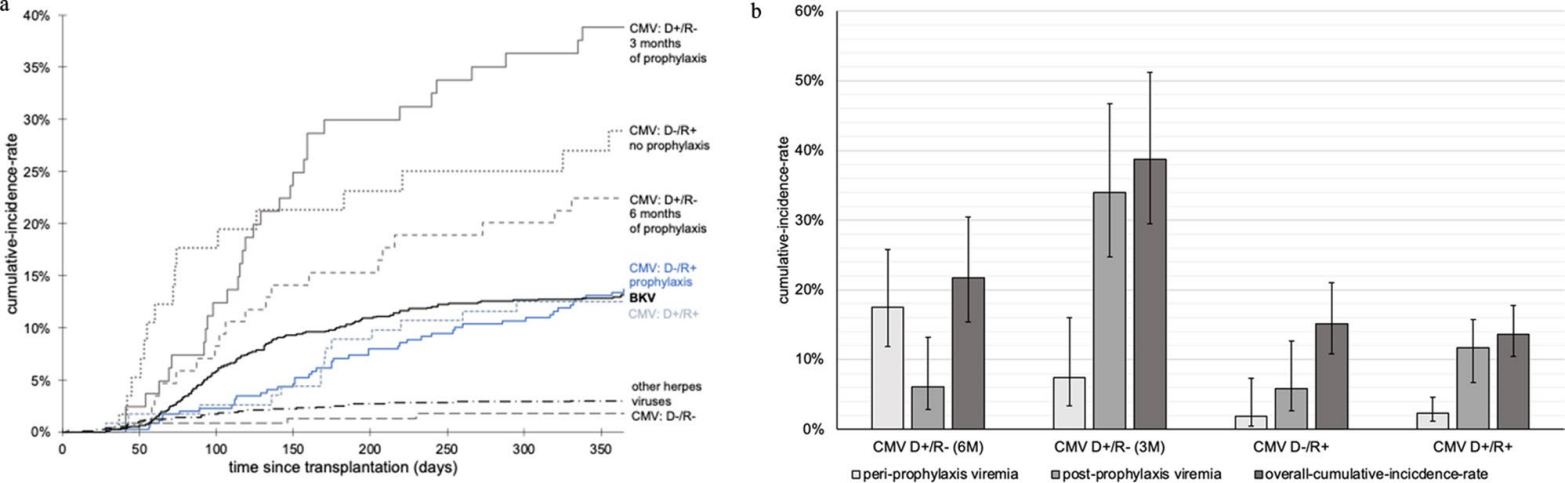
**a**) Cumulative-incidence-rates of CMV, BKV and other herpesviruses. Other herpesviruses included HSV-1 (*n* = 15), HSV-2 (*n* = 6), VZV (*n* = 9), EBV (*n* = 7), HHV-6 (*n* = 1). Abbreviations: CMV=Cytomegalovirus, BKV=BK-virus, HSV = Herpes simplex virus, VZV=Varicella zoster virus, EBV = Epstein-Barr-virus and HHV-6 = Human Herpesvirus 6, D+/- = Donor positive/negative, R+/- = Recipient positive/negative. **b**) Cumulative-incidence-rates of peri- and post-prophylaxis-CMV depending on initial CMV serostatus and prophylaxis duration. Abbreviations: CMV=Cytomegalovirus, D+/- = Donor positive/negative, R+/- = Recipient positive/negative, 6M = 6-month-rophylaxis, 3M = 3-month-prophylaxis

CMV was the predominant agent with an incidence of 14.3% (95% CI, 12.3; 16.6), ranging from 1.8% (95% CI, 0.7; 4.8) in the D−/R − group to 28.6% (95% CI, 22.9; 35.6) in the D+/R − group (Table 2; Fig. 1a). Of all patients with CMV viremia, 13.2% (19/144) experienced more than one episode (average 2.6 episodes per recipient, Md = 75 days, IQR = 46–97) and 5.5% developed an end-organ disease (eight cases of CMV colitis and two cases of CMV pneumonia). End-organ diseases developed later after transplantation (Md = 154 days, IQR = 134–212). and were observed only in recipients who had received a 3-month prophylaxis. CMV syndrome occurred in at least 9.3%. 21.5% of recipients developed CMV syndrome during the prophylaxis period, while 78.4% developed it after the completion of prophylaxis.

Other herpes viruses were rare (Table 2; Fig. 1a), affecting 3.0% (95% CI, 2.2; 4.3) of our cohort. No cases of HHV-7 and HHV-8 were reported. Among the patients affected by EBV, all but one had a positive IgG before transplantation. The one patient with negative IgG (D+/R- status) experienced an early EBV episode, which occurred on day 49 post-transplantation. 28.7% of the EBV-affected patients had received thymoglobuline as induction therapy.

### Breakthrough - and post-prophylaxis CMV viremia

25.9% (45/174) of all CMV viremias were detected within the first 3 months. The median time until onset was 4.5 months (Md = 138 days, IQR = 77–219) after transplantation and 2 months (Md = 59 days, IQR = 0–140) after discontinuation of prophylaxis (Table 2). Of all participants with viremias, 65% (36/103) had viremia after prophylaxis. The incidences of peri- and post-prophylaxis viremia in all risk groups are presented in Fig. 1b.

Breakthrough-prophylaxis viremia was most frequently observed in D+/R − recipients who received 6-month prophylaxis (17.5% [95% CI, 11.8; 25.8]), followed by D+/R − recipients who received 3-month prophylaxis (7.4% [95% CI, 3.4; 16.0]), D-/R + recipients (2.3% [1.2;4.6]) and D+/R + recipients (1.9% [0.5;7.3]). The median time to breakthrough-prophylaxis viremia in these subgroups was 99 days (IQR = 74–132), 60 days (IQR = 50–69), 64 days (IQR = 57–68), and 52 days (IQR = 43–72), respectively.

Post-prophylaxis CMV occurred in 8.4% (95% CI, 6.8; 10.3) of the total cohort, in 34% (95% CI, 24.7; 46.7) of D+/R − recipients who received 3-month-prophylaxis, in 11.7% (95% CI, 8.6; 15.7) of D+/R + recipients, in 6.1% (95% CI, 2.8; 13.2) of D+/R − recipients who received 6-month-prophylaxis, and in 5.8% (95% CI, 2.7; 12.6) of D−/R + recipients. In recipients receiving 3-month prophylaxis, post-prophylaxis viremia occurred the earliest in the D+/R − group (Md = 141 days, IQR = 115–195) and the latest in the D+/R + group (Md = 209 days, IQR = 153–297). Most recipients without prophylaxis experienced early viremia (1.8% in the D−/R − group [95% CI, 0.7; 4.8], median time = 94 days, IQR = 38–167 and 28.9% in the D−/R + group [95% CI, 19.1; 43.8], median time = 95 days, IQR = 56–180).

### Impact of transplant centers’ CMV prophylaxis strategy on virus incidence

Four centers recommended a 6-month CMV prophylaxis for the D+/R- group and a 3-month prophylaxis for the D+/R + and D-/R + groups. One center, recommended a 3-month prophylaxis for all D + patients and no prophylaxis for D- transplant recipients (Supplementary Material, Table S1).

Deviations from center protocols to the guidelines were primarily observed in the D−/R− (34.6% with prophylaxis) and D−/R + group (37.3% without prophylaxis). The latter deviation was due to the complete omission of prophylaxis.

In the D−/R + group, the incidence of CMV viremia was more than doubled in patients not receiving prophylaxis (28.9% [95% CI, 19.1; 43.8] vs. 12.5% [95% CI, 7.7; 20.4], *p* = 0.01) (Fig. 1a), whereas the incidence of leucopenia was halved (7.1% [95% CI, 8.1; 26.9] vs. 14.7% [95% CI, 8.1; 26.9], *p* = 0.25). Providing prophylaxis to the D−/R − group substantially lowered the incidence of other herpes viruses (4.6% [95% CI, 1.5; 13.9] vs. 11.4% [95% CI, 6.9; 18.6], *p* = 0.12), but significantly increased the incidence of leucopenia (20.6% [95% CI, 10.6; 39.8] vs. 2.7% [95% CI, 0.7; 10.7], *p* = 0.03). Overall, the incidence of leucopenia was 14.9% [95% CI, 11.8; 18.9] in patients with prophylaxis and 4.3% [95% CI, 1.8; 10.1] in patients without prophylaxis (*p* = 0.01). The incidences of CMV viremia in the D+/R- group varied widely depending on the duration of CMV prophylaxis, ranging from 21.7% [95% CI, 15.4; 30.5] in patients receiving 6-month-prophylaxis to 38.8% [95% CI, 29.5; 51.2] in patients receiving 3-month-prophylaxis (*p* = 0.03) (Fig. 1a).

### Impact of transplant centers’ BKV monitoring strategy on virus incidence

The incidence of BKV was 13.2% (95% CI, 11.2; 15.4). Of the patients with BKV viremia. 7.7% (13/132) were diagnosed with BKVAN. However, no transplant failure occurred within the first year due to BKVAN. One patient had JCV co-viremia (257.600 copies/ml). No case of progressive multifocal leukoencephalopathy was reported.

Overall, BKV monitoring frequency was below KDIGO guideline recommendations. The incidence of BKV was 20.9% (95% CI, 15.9%; 27.3%) in centers performing a monthly monitoring until month 3 (with fewer or no follow-ups if results remained negative) and 11.2% (95% CI, 9.3; 13.7) in centers conducting routine monitoring monthly till month 3–6 and every 3 months till the end of the first year (*p* < 0.001).

### CMV/BKV-co-viremia

**Fig. 2:**
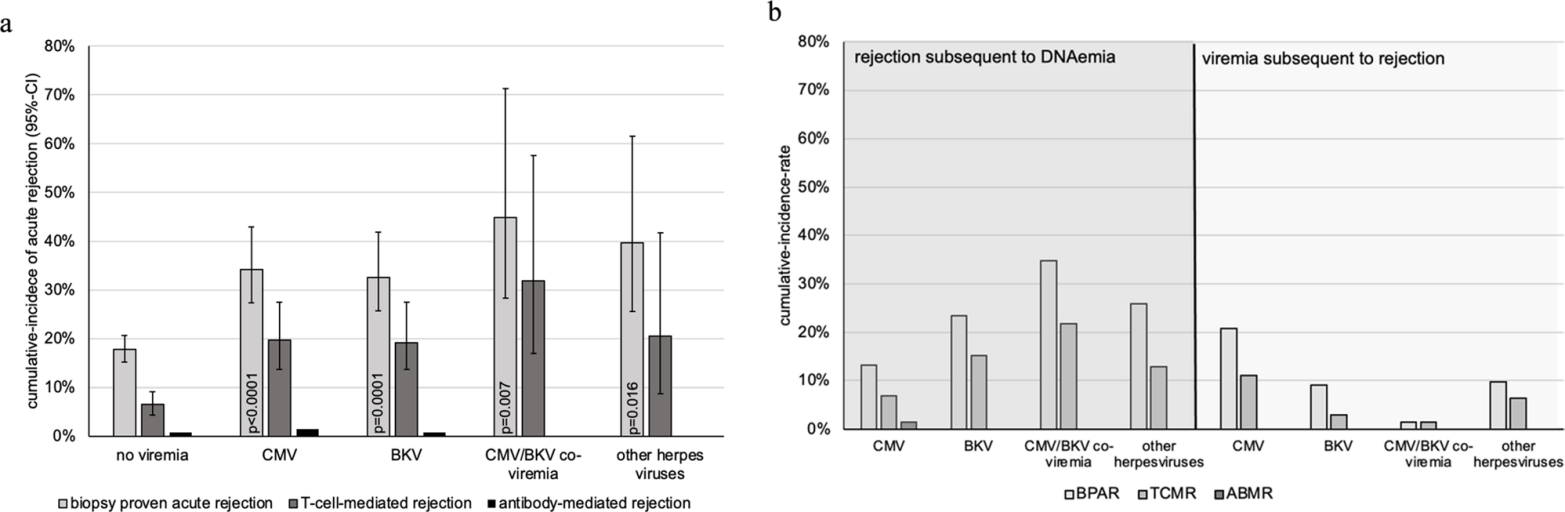
Cumulative-incidence-rates of **a**) acute rejection in viremic and non-viremic patients. Cumulative-incidence-rates were compared to cumulative-incidence-rates of non-viremic patients (CMV-, BKV-, CMV-/BKV-, other herpes viruses-, respectively) using Gray-test. Other herpes viruses included HSV-1 (*n* = 15), HSV-2 (*n* = 6), VZV (*n* = 9), EBV (n = 5), HHV-6 (n = 1). Abbreviations: CMV=Cytomegalovirus, BKV = BK-virus, HSV=Herpes simplex virus, VZV = Varicella zoster virus, EBV = Epstein-Barr-virus and HHV-6 = Human Herpesvirus 6. **b**) infected patients experiencing rejection subsequent to infection (left side) or prior to infection (right side). Abbreviations: BPAR=biopsy proven acute rejection, TCMR=T-cell-mediated acute rejection, ABMR = Antibody-mediated acute rejection

CMV viremia was not associated with an increased risk of BKV viremia (hazard ratio [HR] = 1.064, [95% CI, 0.57; 1.99]) and vice versa (HR = 0.765 [95% CI, 0.34; 1.75], *p* = 0.52). A total of 23 (2.2%) patients had BKV/CMV co-viremia (Table 1). Patients with co-viremia had a notable shorter onset of first viremia than patients with sole CMV or BKV viremia (Md = 85 days vs. Md = 142 days and 109 days, respectively). Most patients with CMV/BKV-co-viremia were male (82.6%), received their allograft from a deceased donor (86.3%), and belonged to the CMV D+/R − group (54.5%). This percentage was notably higher compared to patients with isolated CMV viremia (Table 1). The mean eGFR at 12 months was significantly lower (41.3 ± 18.3 vs. 49.1 ± 19.5 ml/min/1.73m^2^, *p* = 0.03). Acute rejection was observed at least once in 43.5% (10/23) of these patients (Fig. 2a).

### Risk factors for BKV, CMV, other herpes viruses, and CMV-BKV co-viremia

In multivariate analysis, deceased donation (HR = 2.26 [95%CI, 1.44;3.54], *p* < 0.001), CMV D+/R- (HR = 2.40 [95%-CI1.62;3.54], *p* < 0.001), and T-cell-mediated rejection (HR = 3–030 [95%CI, 1.45;6.33], *p* = 0.01) were associated with CMV occurrence. A low eGFR at month 3 (HR = 0.97 [95%CI, 0.97;0.99], *p* < 0.001) and tacrolimus immunosuppression (HR = 1.73 [95%CI, 1.02;2.93], *p* = 0.04) were the risk factors for BKV viremia (Table 3a). CMV D+/R − serostatus was the only independent risk factor for CMV-BKV co-viremia (HR = 3.06 [95%CI, 1.14;8.21], *p* = 0.03) (Table 3a). The occurrence of other herpes viruses was independently associated with the non-administration of CMV prophylaxis (HR = 0.22 [95%CI, 0.11;0.47], *p* < 0.001) and with a preceding fungal infection (HR = 5.44 [95%CI, 1.63;18.11], *p* = 0.01) (Table 3b) Further analysis indicated that viremias associated with preceding fungal infections were primarily caused by HSV-1 and VZV, whereas EBV viremias were observed prior to the onset of fungal infections (Supplementary Material, Table S2).

**Table 3a:** Risk factors for CMV and BKV viremia. Analyses were performed using Cox regression proportional hazards analysis. Covariates with a *p* < 0.100 were included in multivariate analyses

	CMV						BKV					
	Univariate			Multivariate			Univariate			Multivariate		
	HR	95%-CI	*p*	HR	95%-CI	*p*	HR	95%-CI	*p*	HR	95%-CI	*p*
**Baseline data**												
Age (years)	0.998	0.987;1.010	0.765				1.007	0.995;1.020	0.268			
Male gender	1.028	0.729;1.448	0.867				**1.577**	**1.071;2.321**	**0.021**	1.446	0.958;2.184	0.079
Deceased donor	**2.004**	**1.340;2.997**	**< 0.001**	**2.258**	**1.441;3.539**	**< 0.001**	1.119	0.773:1.618	0.552			
ESP	0.720	0.415;1.249	0.243				0.654	0.361;1.184	0.161			
Body mass index (kg/m^2^)	1.000	0.964;1.037	0.981				0.990	0.951;1.030	0.603			
Previous transplantation	0.983	0.629;1.535	0.939				1.181	0.759:1.838	0.462			
Pancreas-kidney	**1.707**	**0.945;3.083**	**0.076**	1.449	0.755;3.2.781	0.162	0.523	0.193;1.416	0.202			
AB0i	0.692	0.306;1.569	0.378				0.750	0.331;1.702	0.492			
CMV D+/R-	**3.047**	**2.169;4.280**	**< 0.001**	**2.396**	**1.620;3.544**	**< 0.001**	1.318	0.888;1.957	0.170			
CMV R+	0.961	0.690;1.339	0.815				**0.712**	**0.506;1.004**	**0.053**	0.759	0.525;1.098	0.143
CMV D+	**2.525**	**1.715;3.718**	**< 0.001**	1.367	0.825;2.265	0.226	1.006	0.712;1.423	0.972			
CMV prophylaxis^1^	**2.815**	**1.648;4.807**	**< 0.001**	1.607	0.836;3.090	0.155	0.841	0.573;1.233	0.375			
Age class donor^2^	1.040	0.985;1.098	0.153				1.038	0.982;1.097	0.190			
Delayed graft function^3^	1.272	0.859;1.884	0.230				0.824	0.517;1.315	0.418			
**Initial immunosuppression**												
Conditioning treatment^4^	**1.493**	**1.064;2.094**	**0.020**	1.500	0.856;2.630	0.157	1.041	0.716;1.513	0.833			
Plasmapheresis	**1.658**	**1.103;2.492**	**0.016**	1.472	0.824;2.630	0.192	1.303	0.824;2.050	0.258			
Thymoglobuline vs. BSX	**1.620**	**1.103;2.380**	**0.014**	0.907	0.513;1.605	0.737	1.209	0.782;1.869	0.392			
Tacrolimus vs. Csa	1.210	0.771;1.897	0.407				**1.640**	**0.968;2.778**	**0.066**	**1.726**	**1.016;2.934**	**0.044**
**Other events within first year posttransplantation**												
BPAR	**1.760**	**1.162;2.658**	**0.007**	0.998	0.560;1.776	0.994	0.738	0.407;1.340	0.319			
TCMR	**2.831**	**1.704;4.703**	**< 0.001**	**3.030**	**1.451;6.325**	**0.003**	0.803	0.296;2.179	0.666			
Bacterial infection	**1.546**	**1.108;2.155**	**0.010**	1.298	[0.919;1.833]	0.139	0.891	0.284;2.802	0.844			
Fungal infection	1.585	0.699;3.592	0.270				0.927	0.641;1.343	0.690			
**Renal function 3 months after transplantation**												
eGFR in ml/min/1.73m^2^	0.998	0.989;1.006	0.573				**0.978**	**0.969;0.987**	**< 0.001**	**0.977**	**0.968;0.987**	**< 0.001**

Abbreviations: CMV = Cytomegalovirus, BKV = BK-virus, ESP = Eurotransplant Senior Program, BSX = Basiliximab, Csa = Ciclosporine A, BPAR = biopsy proven acute rejection, TCMR = T-cell-mediated rejection, eGFR = estimated glomerular filtration rate, D+/-=Donor positive/negative, R+/-=Recipient positive/negative; HR = Hazard ratio, 95%-CI = 95%-confidence-interval

^1^ Valganciclovir, ^2^ In 5-year intervals, ^3^ Need for hemodialysis within the first 7 days post-transplantation, ^4^ Rituximab, immunadsorption, plasmapheresis or thymoglo for induction therapy;

**Table 3b:** Risk factors for other herpes viruses and combined CMV/BKV viremia. Analyses were performed using Cox regression proportional hazards analysis. Covariates with a *p* < 0.100 were included in multivariate analyses

	Other herpes viruses (*n* = 31)						CMV/BKV co-viremia (*n* = 23)					
	Univariate			Multivariate			Univariate			Multivariate		
	HR	95%-CI	*p*	HR	95%-CI	*p*	HR	95%-CI	*p*	HR	95%-CI	*p*
**Baseline data**												
Age (years)	1.013	0.986;1.039	0.353				0.994	0.965;1.023	0.670			
Male gender	1.588	0.710;3.551	0.260				**2.619**	**0.891;7.698**	**0.080**	2.422	0.811;7.230	0.113
Deceased donor	0.912	0.437;1.904	0.807				**3.217**	**0.952;10.870**	**0.060**	3.043	0.886;10.451	0.077
ESP	0.922	0.347;2.834	0.987				0.303	0.041;2.246	0.243			
Body mass index (kg/m^2^)	0.971	0.893;1.055	0.481				0.975	0.885;1.074	0.606			
Previous transplantation	1.246	0.511;3.037	0.629				1.446	0.537;3.894	0.466			
Pancreas-kidney	0.573	0.078;4.201	0.587				0.788	0.106;5.849	0.816			
AB0i	0.548	0.075;4.021	0.554				0.760	0.102;5.651	0.789			
CMV D+/R-	**0.034**	**0.001;1.460**	**0.078**	-	-	-	**4.619**	**1.996;10.691**	**< 0.001**	**3.062**	**1.142;8.212**	**0.026**
CMV R+	0.580	0.284;1.184	0.134				0.558	0.239;1.306	0.179			
CMV D+	**0.189**	**0.077;0.462**	**< 0.001**	0.999	0.945;1.056	0.972	**3.514**	**1.189;10.383**	**0.023**	1.736	0.396;7.614	0.464
CMV prophylaxis^1^	**0.207**	**0.100;0.430**	**< 0.001**	**0.224**	**0.107;0.469**	**< 0.001**	**3.395**	**0.796;14.481**	**0.099**	1.205	0.199;7.285	0.724
Age class donor^2^	1.044	0.929;1.174	0.469				1.074	0.933;1.236	0.320			
Delayed graft function^3^	1.274	0.549;2.957	0.573				0.196	0.026;1.456	0.111			
**Initial immunosuppression**												
Conditioning treatment^4^	1.707	0.836;3.484	0.142				1.524	0.669;2.530	0.324			
Plasmapheresis	0.672	0.204;2.211	0.513				**2.270**	**0.895;5.757**	**0.084**	2.596	0.944;7.143	0.065
Thymoglobuline vs. BSX	1.466	0.632;3.402	0.373				1.778	0.701;4.510	0.226			
Tacrolimus vs. Csa	0.591	0.271;1.291	0.187				2.172	0.502;9.403	0.299			
**Other events within first year post transplantation**												
Acute biopsy-proven rejection	1.330	0.458;3.855	0.600				1.261	0.370;4.299	0.711			
TCMR	2.160	0.503;9.271	0.300				1.356	0.179;10.242	0.768			
Bacterial infection	1.593	0.765;3.315	0.213				0.779	0.306;1.986	0.601			
Fungal infection	**4.732**	**1.432;15.637**	**0.011**	**5.443**	**1.636;18.111**	**0.006**	0.048	0.000;4.367	0.602			
**Renal function 3 months after transplantation**												
eGFR in ml/min/1.73m^2^	**0.981**	**0.958;1.003**	**0.094**	0.983	0.961;1.005	0.132	0.998	0.989;1.006	0.573			

Abbreviations: CMV = Cytomegalovirus, BKV = BK-virus ESP = Eurotransplant Senior Program,, BSX = Basiliximab, Csa = Ciclosporine A, BPAR = biopsy proven acute rejection, TCMR= -cell-mediated rejection, eGFR = estimated glomerular filtration rate, D+/-= Donor positive/negative, R+/-=Recipient positive/negative; HR = Hazard ratio, 95%-CI = 95%-confidence-interval

Other herpes viruses included HSV-1 (*n* = 15), HSV-2 (*n* = 6), VZV(*n* = 9), EBV (*n* = 5) and HHV-6 (*n* = 1).

^1^ Valganciclovir, ^2^ In 5-year intervals, ^3^ Need for hemodialysis within the first 7 days post-transplantation, ^4^ Rituximab, immunadsorption, plasmapheresis or thymoglobuline for induction therapy

### Risk factors for death, graft loss and fungal infections

For death, significant risk factors included participation in the Eurotransplant Senior Program (ESP) (HR: 1.33, [95% CI, 1.31;10.81], *p* = 0.014), T-cell-mediated rejection (HR: 4.39, [95% CI, 1.53;12.61], *p* = 0.006), and fungal infections (HR: 5.90, [95% CI, 1.88;18.50], *p* < 0.001). Graft loss was significantly associated with ESP participation (HR: 3.14, [95% CI, 1.32;7.50], *p* = 0.010) and TCMR (HR: 4.70, [95% CI, 1.99;11.12.71], *p* = 0.001). In terms of fungal infections, independent risk factors included ESP participation (HR: 3.80, [95% CI, 2.02;7.17], *p* < 0.001), pancreas-kidney transplantation (HR: 3.85, [95% CI, 1.69;8.79], *p* = 0.001), and EBV infection (HR: 12.60, 95% CI: 3.82;41.55, *p* < 0.001) (Supplementary Material, Table S2).

### Outcome of herpes- and polyomavirus viremia

CMV viremia was associated with a higher risk of bacterial infection (HR = 1.77, [95% CI, 1.03; 3.02], *p* = 0.04). Other herpes viruses were associated with a quadrupled risk for fungal infection (HR = 4.34, [95% CI, 1.03; 18.30], *p* = 0.04). The incidence of fungal infection in patients with other herpesviruses was 19.7% (95% CI, 9.6; 40.2) compared with 4.2% (95% CI, 3.1; 5.7) in the remaining cohort. The incidence was highest in patients with EBV (42.9%). BKV viremia was associated neither with a higher risk for bacterial infection (HR = 0.98, [95% CI, 0.74;1.31], *p* = 0.91) nor with a higher risk for fungal infection (HR = 1.17, [95% CI, 0.53; 2.62], *p* = 0.70).

Patient and allograft survival in the viremic cohort was not different from that in the non-viremic cohort (*p* = 0.36, *p* = 0.55, respectively), but incidence of acute rejection was significantly higher in the viremic cohort (31.9% [95% CI, 26.8; 38.0] vs. 17.6% [95% CI, 15.1; 20.6]; *p* < 0.001), with almost triple the incidence of T-cell-mediated rejection (18.0% [95% CI, 14.0; 23.3] vs. 6.5% [95% CI, 5.0; 8.6], *p* < 0.001). Of note, over half of CMV viremia episodes occurred after acute rejection and its treatment (Md = 90 days, IQR = 58–162). CMV/BKV co-viremic patients were most commonly affected by acute rejections (43.5%, Fig. 2a), mainly after experiencing viremia (Fig. 2b). Of these 10 patients, five (50%) received an intensified induction therapy (plasmapheresis and thymoglobuline), four because of immunization and one because of AB0 incompatibility.

## Discussion

Despite prophylaxis recommendations, 27% of our large multicenter cohort of kidney transplant recipients developed herpes or polyomavirus viremia to a level consistent with active infection within the first year after transplantation. Consistent with previous findings, CMV and BKV were the predominant agents, with the highest CMV-incidence observed in D+/R − recipients [20, 21]. In the KDIGO 2009 guidelines a CMV prophylaxis with oral ganciclovir or valganciclovir has been recommended for at least 3-months after transplantation, except when donor and recipients both have negative CMV serologies [14]. In the present study, most recipients with post-prophylaxis viremia received a 3-month-regimen, suggesting effectiveness while administered. A 6-month-regimen reduced viremia incidence in D+/R − recipients by nearly 50%, consistent with the IMPACT trial, which showed that extending prophylaxis to 200 days significantly lowered CMV rates compared to 100 days [22], despite debates over the trial’s design and execution [23]. Although current guidelines as the recently published German S2k guideline „virus infection in organ transplantation“ endorse extended prophylaxis for D+/R − recipients [24], adherence to these recommendations in clinical practice varies and factors such as an more individualized risk assessment, patient adherence are often overlooked.

A critical aspect influencing prophylaxis efficacy is the accuracy of antiviral dosing. Breakthrough viremias suggest potential underdosing due to renal function adjustments. While prescribing information recommends dosing based on the Cockcroft-Gault formula, clinical practice often defaults to CKD-EPI, increasing the risk of underdosing or overdosing, leading to inadequate viral suppression, resistance [25] or heightened toxicity, such as leukopenia [26]. In our cohort, valganciclovir nearly tripled the incidence of leukopenia. Leukopenia may lead to reduction of the immunosuppression followed by rejection episodes. Letermovir, a novel prophylactic agent offers fewer side effects but is costly, might delay CMV-specific immune reconstitution and lacks efficacy against other herpesviruses [27, 28]. In current clinical guidelines it is only recommended as an alternative to valganciclovir in D+/R − recipients [24].

Given the frequent onset of CMV viremia observed post-rejection, the six-week prophylaxis duration suggested by KDIGO may be inadequate for specific patient groups emphasizing the need for extended risk assessment to better tailor prophylaxis or monitoring. Additional monitoring may also be beneficial for recipients of deceased donor grafts undergoing intensified induction therapy. However, the efficacy and feasibility of such a hybrid strategy remain uncertain due to the lack of reliable data [24]. Alternative monitoring strategies have been investigated [29–32], including monitoring of CMV-specific T-cell-mediated-immunity to individualize the duration of prophylaxis thereby preventing further prophylaxis once sufficient immunity is reached [33, 34].

Our results also indicated that D−/R − individuals without prophylaxis were at increased risk for other herpesvirus infections. The current recommendation to omit prophylaxis for D−/R − recipients [14] may oversimplify risk assessment. In particular, HSV-seronegative transplant recipients represent a high-risk group for severe HSV infections [35]. Other herpesviruses were also associated with a higher incidence of fungal infections, with a notable clustering observed after EBV infection. However, due to the low number of EBV cases and the observational nature of the study, drawing conclusions remains challenging. As demonstrated by our results, invasive fungal infections continue to be a major cause of death in kidney recipients. The presence of viral infections may further impair immune function [36–39], potentially exacerbating each other and compounding the risk of worse clinical outcomes. This underscores the need for heightened attention to co- or secondary infections, especially in patients with additional risk factors.

Despite the limitations of existing guidelines, our results showed, adherence to KDIGO prophylaxis recommendations could significantly lower current incidences, particularly in CMV D−/R + patients, 37.2% of whom did not receive prophylaxis, doubling viremia rates. Preemptive antiviral therapy should be recommended in CMV D−/R + patients if prophylaxis is not administered. The VIPP trial demonstrated that prophylaxis is more effective than a preemptive approach in preventing CMV infection and disease in intermediate-risk patients, though both strategies were similarly effective in preventing graft loss and death [40].

Another key finding is that BKV monitoring was performed less frequently than recommended across all centers, suggesting an opportunity to mitigate the burden of significant BKV viremia. Current guidelines even recommend monthly monitoring for the first 9 months post-transplant, followed by quarterly monitoring up to 2 years. However, it remains a “one-size-fits-all” approach and may not adequately address individual risk profiles [6]. Pre-transplant measurement of BKV-specific IgG might facilitate risk stratification, allowing for a more efficient monitoring [6, 41]. Despite its potential, its routine clinical use is hindered by the lack of standardized assays and limited commercial availability [6].

We did not observe a higher risk of death or allograft loss in patients with herpes or polyomavirus viremia, possibly due to the limited one-year follow-up. Allograft function was significantly impaired one year after transplantation and acute rejections were significantly more prevalent, reflecting challenges in balancing over- and underimmunosuppression. Most rejections occurred post-infection, likely due to reduced immunosuppression, though viral replication-induced inflammation may also disrupt graft tolerance [42–44]. Cell-therapy protocols show promise in reducing rejection rates and infection-related side effects [45, 46].

Our study is the first to highlight the impact of non-adherence to prophylaxis and monitoring guidelines by transplant centers, that has previously been completely overlooked. Although our results provide insights into routine transplant care in Germany, their exploratory nature requires cautious interpretation as we cannot prove causal relationships. Variability in adherence across transplant centers, differences in PCR cutoffs, and individual physician decisions limit generalizability. In addition, patient adherence to prophylaxis was not assessed, and the lack of data on valganciclovir dosing limits conclusions on breakthrough infections.

## Conclusion

Herpes- and polyomaviruses continue to be a significant challenge after renal transplantation, with their incidence being strongly influenced by inconsistent adherence to prophylaxis and monitoring guidelines across transplant centers in real-world clinical practice. This newly recognized issue adds to the existing weaknesses of the guidelines. Standardizing documentation of patient adherence and transplant center practices would enable a more thorough evaluation of guideline adherence and outcomes. Future guidelines should focus on differentiated risk stratifications. For CMV, extended monitoring and individualized prophylaxis duration are necessary to prevent post-prophylaxis or post-rejection viremia, particularly in high-risk groups. For BKV, guidelines should establish risk-adapted monitoring schemes. Additionally, guidelines should include protocols for early detection and management of co- and secondary infections, especially fungal infections, refine immunosuppression adjustments during infections, and suggest tailored prophylaxis for D−/R − subgroups to prevent herpesvirus infections beyond CMV.

## Electronic supplementary material

Below is the link to the electronic supplementary material.

Supplementary Material 1

## Data Availability

The data that support the findings of this study are available from DZIF Transplant Cohort e.V. but restrictions apply to the availability of these data, which were used under license for the current study, and so are not publicly available. Data are however available from the corresponding author upon reasonable request and with permission of DZIF transplant cohort e.V.

## Abbreviations

ABMR: Antibody-mediated rejection
APKD: Autosomal polycystic kidney disease
ATG: Antithymoglobulin
BSX: Basiliximab BKV = BK-virus
BKVAN: BKV-associated nephropathy
BPAR: Biopsy proven acute rejection
C0: Trough level
Csa: Ciclosporine A
CMV: Cytomegalovirus
DZIF: German Center for Infection Research D+/-=Donor positive/negative
DSA: Donor specific antibodies
EBV: Epstein-Barr-virus
ESP: Eurotransplant Senior Program
eGFR: Estimated glomerular filtration rate
ESRD: End-stage renal disease
KDIGO: Kidney Disease: Improving Global Outcome
HHV-6: Human Herpesvirus 6
HHV-7: Human Herpesvirus 7
HHV-8: Human Herpesvirus 8
HLA: anti-human leukocyte antigen
HR: Hazard ratio
HSV: Herpes simplex virus
IQR: Interquartile range JCV = JC-Virus
md: Median
MMF: Mycophenolate mofetil
MPA: Mycophenolic acid
no.: Number
R+/-: Recipient positive/negative
SD: Standard deviation
Tac: Tacrolimus
TCMR: T-cell-mediated rejection
VZV: Varicella zoster virus
95%-CI: 95%-confidence-interval
