# Supplementary material for: Transplant centers’ prophylaxis and monitoring strategies: a key determinant of current herpes and polyomavirus incidences – results from the DZIF kidney transplant cohort
Source: BMC Nephrol. 2025 Apr 30;26:218. doi: 10.1186/s12882-025-04084-5 (PMC12045003; doi:10.1186/s12882-025-04084-5)
Supplement: Supplementary file 1 — Supplementary Material 1 [file 12882_2025_4084_MOESM1_ESM.docx]

**Supplementary Material**

Table S1. Centers’ standard on CMV prophylaxis and routine CMV, BKV, EBV monitoring during the first year after transplantation.

|  | **Center 1** | **Center 2** | **Center 3** | **Center 4** | **Center 5** |
| --- | --- | --- | --- | --- | --- |
| **CMV prophylaxis (valganciclovir)** | | | | | |
| D+/R- | 3 months | 6 months | 6 months | 6 months | 6 months |
| D+/R+ | 3 months | 3 months | 3 months | 3 months | 3 months |
| D-/R+ | no | 3 months | 3 months | 3 months | 3 months |
| D-/R- | no | no | no | no | no |
| immunized (T cell depleting induction) | 3 months | 3 months | 3 months | 3 months | 3 months |
| **Screening** | | | | | |
| CMV | Indication-related | Weekly for 3 months*, once per month thereafter | Weekly for 3 months*, every 3 months thereafter | Indication-related | Week 1, 2, (3)^#^; indication-related thereafter |
| BKV (blood) | Monthly within the first 6 months, month 9, 12 | Weekly for 3 months*, indication-related thereafter | Monthly within the first 3 to 6 months, indication-related thereafter | Monthly within the first 6 months, month 9, 12 | Every 3 months within the first year |
| EBV | no | Indication-related | no | In case of EBV seronegative recipient | Indication-related |
| Abbreviations: CMV = Cytomegalovirus, D+/- = Donor positive/negative, R+/- = Recipient positive/negative,, EVV = Epstein-Barr virus  *only if visits are performed in the transplant center  ^#^ weekly during the first inpatient stay | | | | | |

Table S2. Risk factors for death, graft loss and fungal infections

|  | **death** | | | | | | **graft loss** | | | | | |
| --- | --- | --- | --- | --- | --- | --- | --- | --- | --- | --- | --- | --- |
|  | **univariate** | | | **multivariate** | | | **univariate** | | | **multivariate** | | |
|  | **HR** | **95%-CI** | **p** | **HR** | **95%-CI** | **p** | **HR** | **95%-CI** | **p** | **HR** | **95%-CI** | **p** |
| **Baseline data** |  |  |  |  |  |  |  |  |  |  |  |  |
| age (years) | **1.084** | **1.027;1.143** | **0.003** |  |  |  | **1.080** | **1.037;1.124** | **<0.001** |  |  |  |
| male gender | 1.116 | 0.478;2.607 | 0.800 |  |  |  | 1.099 | 0.470;2.568 | 0.827 |  |  |  |
| deceased donor | - | - | 0.996 |  |  |  | **5.514** | **1.297;23.450** | **0.021** |  |  |  |
| ESP | **3.390** | **1.451;7.922** | **0.005** | **1.325** | **1.309;10.808** | **0.014** | **3.292** | **1.409;7.693** | **0.006** | **3.140** | **1.315;7.499** | **0.010** |
| body mass index (kg/m^2^) | **1.085** | **1.011;1.164** | **0.024** | 1.069 | 0.959;1.192 | 0.230 | 1.001 | 0.961;1.095 | 0.978 |  |  |  |
| previous transplantation | 2.607 | 0.979;6.947 | 0.055 |  |  |  | 0.466 | 0.110;1.980 | 0.301 |  |  |  |
| pancreas-kidney | 2.219 | 0.510;9.653 | 0.288 |  |  |  | - | - | 0.995 |  |  |  |
| AB0i | - | - | 0.995 |  |  |  | 0.726 | 0.098;5.388 | 0.754 |  |  |  |
| CMV D+/R- | 1.081 | 0.356;3.285 | 0.891 |  |  |  | 0.542 | 0.162;1.819 | 0.322 |  |  |  |
| CMV R+ | 1.616 | 0.607;4.306 | 0.337 |  |  |  | 1.129 | 0.502;2.542 | 0.769 |  |  |  |
| CMV D+ | 2.724 | 0.897;8.276 | 0.077 |  |  |  | 0.654 | 0.293;1.459 | 0.300 |  |  |  |
| CMV prophylaxis^1^ | 0.6780 | 0.090;5.094 | 0.706 |  |  |  | 0.420 | 0.099;1.792 | 0.241 |  |  |  |
| age class donor^2^ | **6.157** | **1.871;20.257** | **0.003** |  |  |  | **2.621** | **1.208;5.684** | **0.015** |  |  |  |
| delayed graft function^3^ | **3.674** | **1.646,8.201** | **0.001** | 2.020 | 0.694;5.874 | 0.197 | 3.666 | 1.643;8.184 | 0.002 |  |  |  |
| **initial immunosuppression** | | | | | | | | | | | | |
| conditioning treatment^4^ | 0.672 | 0.221;2.042 | 0.483 |  |  |  | 0.832 | 0.328;2.111 | 0.699 |  |  |  |
| Plasmapheresis | 1.263 | 0.366;4.363 | 0.712 |  |  |  | 0.903 | 0.269;3.028 | 0.869 |  |  |  |
| Thymoglobuline vs. BSX | 1.190 | 0.247;5.729 | 0.828 |  |  |  | 0.713 | 0.213;2.390 | 0.583 |  |  |  |
| Tacrolimus vs. Csa | 0.893 | 0.186;4.301 | 0.888 |  |  |  | 0.596 | 0.229;1.551 | 0.289 |  |  |  |
| **other events within first year posttransplantation** | | | | | | | | | | | | |
| BPAR | **3.719** | **1.305;10.603** | **0.014** |  |  |  | **2.734** | **1.214;6.154** | **0.015** |  |  |  |
| TCMR | **7.339** | **2,546;21.152** | **<0.001** | **4.390** | **1.529;12.607** | **0.006** | **5.056** | **2.164;11.817** | **<0.001** | **4.703** | **1.988;11.121** | **<0.001** |
| CMV | 1.226 | 0.355;4.234 | 0.748 |  |  |  | 0.554 | 0.130;2.355 | 0.424 |  |  |  |
| HSV-1 | 3.998 | 0.532;30.043 | 0.178 |  |  |  | 3.000 | 0.405;22.217 | 0.282 |  |  |  |
| HSV-2 | - | - | 0.996 |  |  |  | **7.950** | **1.073;58.881** | **0.042** | 1.857 | 0.240;14.392 | 0.554 |
| VZV | - | - | 0.996 |  |  |  | 5.296 | 0.715;39.218 | 0.103 |  |  |  |
| EBV | - | - | 0.996 |  |  |  | 6.278 | 0.848;46.482 | 0.072 | 7.206 | 0.948;54.787 | 0.056 |
| BKV | 0.397 | 0.053;2.985 | 0.370 |  |  |  | 0.293 | 0.040;2.169 | 0.229 |  |  |  |
| bacterial infection | 2.229 | 0.864;5.749 | 0.097 |  |  |  | 1.995 | 0.886;4.491 | 0.095 |  |  |  |
| fungal infection | **11.059** | **4.150;29.472** | **<0.001** | **5.900** | **1,881;18.502** | **0.002** | 0.889 | 0.120;6.583 | 0.908 |  |  |  |
|  |  | | | | | |  |  |  |  |  |  |
|  |  | | | | | |  |  |  |  |  |  |
|  |  | | | | | |  |  |  |  |  |  |
|  |  | | | | | |  |  |  |  |  |  |
|  |  | | | | | |  |  |  |  |  |  |
|  | **fungal infection** | | | | | |  |  |  |  |  |  |
|  | **univariate** | | | **multivariate** | | |  |  |  |  |  |  |
|  | **HR** | **95%-CI** | **p** | **HR** | **95%-CI** | **p** |  |  |  |  |  |  |
| **Baseline data** |  |  |  |  |  |  |  |  |  |  |  |  |
| age (years) | **1.034** | **1.010,1.058** | **0.004** |  |  |  |  |  |  |  |  |  |
| male gender | 0.730 | 0.410;1.302 | 0.287 |  |  |  |  |  |  |  |  |  |
| deceased donor | **3.586** | **1.524;8.453** | **0.003** |  |  |  |  |  |  |  |  |  |
| ESP | **3.059** | **1.661;5.663** | **<0.001** | **3.803** | **2.018;7.169** | **<0.001** |  |  |  |  |  |  |
| body mass index (kg/m^2^) | 1.007 | 0.946;1.071 | 0.836 |  |  |  |  |  |  |  |  |  |
| previous transplantation | 1.202 | 0.582;2.481 | 0.691 |  |  |  |  |  |  |  |  |  |
| pancreas-kidney | **3.185** | **1.429;7.100** | **0.005** | **3.852** | **1.688;8.791** | **0.001** |  |  |  |  |  |  |
| AB0i | 0.344 | 0.047;2.497 | 0.292 |  |  |  |  |  |  |  |  |  |
| CMV D+/R- | 1.161 | 0.591;2.281 | 0.664 |  |  |  |  |  |  |  |  |  |
| CMV R+ | 0.953 | 0.540:1-681 | 0.867 |  |  |  |  |  |  |  |  |  |
| CMV D+ | 1.255 | 0.697;2.260 | 0.449 |  |  |  |  |  |  |  |  |  |
| CMV prophylaxis^1^ | 1.791 | 0.247;13.002 | 0.564 |  |  |  |  |  |  |  |  |  |
| age class donor^2^ | 1.290 | 0.815;2.042 | 0.277 |  |  |  |  |  |  |  |  |  |
| delayed graft function^3^ | 3.995 | 2.252;7.085 | <0.001 |  |  |  |  |  |  |  |  |  |
| **initial immunosuppression** | | | | | | |  |  |  |  |  |  |
| conditioning treatment^4^ | 1.286 | 0.712;2.323 | 0.405 |  |  |  |  |  |  |  |  |  |
| Plasmapheresis | 1.059 | 0.475;2.361 | 0.888 |  |  |  |  |  |  |  |  |  |
| Thymoglobuline vs. BSX | 1.477 | 0.754;2.896 | 0.256 |  |  |  |  |  |  |  |  |  |
| Tacrolimus vs. Csa | 2.591 | 0.927;7.240 | 0.069 |  |  |  |  |  |  |  |  |  |
| **other events within first year posttransplantation** | | | | | | |  |  |  |  |  |  |
| BPAR | 0.793 | 0.371;1.693 | 0.548 |  |  |  |  |  |  |  |  |  |
| TCMR | 1.183 | 0.468;2.986 | 0.723 |  |  |  |  |  |  |  |  |  |
| CMV | 0.736 | 0.291;1.857 | 0.516 |  |  |  |  |  |  |  |  |  |
| HSV-1 | 1.705 | 0.235;12.356 | 0.698 |  |  |  |  |  |  |  |  |  |
| HSV-2 | - | - | 0.994 |  |  |  |  |  |  |  |  |  |
| VZV | - | - | 0.993 |  |  |  |  |  |  |  |  |  |
| EBV | **10.656** | **3.308;34.319** | **<0.001** | **12.601** | **3.821;41.550** | **<0.001** |  |  |  |  |  |  |
| BKV | 1.17 | 0.528;2.619 | 0.070 |  |  |  |  |  |  |  |  |  |
| ^1^ Valganciclovir, ^2^ age classes: < 35 years, 55-60 years, > 60 years ^3^ Need for hemodialysis within the first 7 days post-transplantation, ^4^ Rituximab, immunadsorption, plasmapheresis or thymoglobuline for induction therapy; Abbreviations: ESP = Eurotransplant Senior Program, BSX = Basiliximab, Csa = Ciclosporine A, BPAR=biopsy proven acute rejection, TCMR =T -cell-mediated rejection, D+/- = Donor positive/negative, R+/- = Recipient positive/negative, CMV = Cytomegalovirus, HSV = Herpes simplex virus, VZV = Varicella zoster virus, EBV = Epstein Barr Virus, BK = BK-virus; HR = Hazard ratio, 95%-CI=95%-confidence-interval | | | | | | |  |  |  |  |  |  |
